# Sustainability logos and claims on food packaging and labels: The case of Türkiye

**DOI:** 10.1371/journal.pone.0337813

**Published:** 2025-12-02

**Authors:** Nesli Ersoy, Bensu Budak

**Affiliations:** Department of Nutrition and Dietetics, Faculty of Health Sciences, Hacettepe University, Ankara, Turkey; Necmettin Erbakan Üniversitesi: Necmettin Erbakan Universitesi, TÜRKIYE

## Abstract

Enhancing consumer awareness of sustainable nutrition is a key factor in promoting public health and supporting environmentally friendly food systems. Logos and claims on food packaging that indicate the use of sustainable production methods have the potential to increase awareness, improve accessibility, and encourage producers. This study represents the first systematic audit aimed at assessing the current situation in Türkiye concerning the use of sustainability-related labels and claims, while also providing scientific insights for producers. The presence of sustainability-related logos and claims was examined on food packages displayed in the shelves of one branch from each major grocery chain in a district of Türkiye’s Region 1. In total, 3,085 food products were assessed in three grocery stores. The analysis included logos and claims related to sustainability, such as animal welfare, organic production, recyclable packaging, the Green Dot (ÇEVKO), Rainforest Alliance certification, local production, geographical indication, good agricultural practices, and vegan/vegetarian declarations. All grocery stores provide recycling bins, and reusable shopping bags are strategically positioned in highly visible areas to encourage consumer use. It has been determined that the logo most frequently appearing on food packaging is the recycling logo (87.4%), while the least frequently appearing logos are the organic production logo (0.3%) and the animal welfare declaration (0.13%). The food categories displaying the greatest number of logos on their packaging, in order, were beverages, sugars (including candies), and meat, poultry, fish, and their products. There is a need to increase the number and visibility of sustainability-related logos and claims on food products. The food industry should be encouraged to adopt more environmentally sustainable practices, both in production and packaging.

## Introduction

Sustainability is a social concept that includes topics such as biodiversity loss, climate change, water pollution, unsustainable water use and the maintenance of the world population with limited resources [1]). In addition to areas such as technology and energy use, sustainability also extends to the fields of health and food consumption. Sustainability is fundamentally intertwined with the food system, as each stage (from production and processing to distribution and consumption) influences key environmental outcomes, including greenhouse gas emissions, land and water use, and biodiversity loss [2,3].

Sustainable food labelling captures the ecological footprint of food products, while the broader concept of sustainable food production underscores the need to operate within planetary boundaries to protect environmental resources and ecosystems [4,5]. In particular, sustainability-related food labels increase transparency by reducing the information imbalance that currently exists along the food chain between food chain stakeholders (e.g., producers, retailers) and consumers, and by informing consumers in a way that can encourage sustainable consumption [6,7].

Labels identify products and provide consumers with accessible information at the point of purchase, thereby playing an important role in shaping food selection, purchasing, and consumption behaviors [8–10]. There are studies indicating that food labels and claims are also effective in terms of health indicators [11,12]. Simultaneously, consumers are presented with increasingly environmentally friendly and ethically produced options, empowering them to make informed choices [1,13]. One way to change individuals’ dietary choices could be through the use of sustainability labels that provide environmental sustainability information on foods may motivate sustainable food selection, purchase or consumption. The effectiveness of sustainability labels depends on whether customers pay attention to them [7]. However, it’s worth noting that, despite increased consumer awareness in recent years, sustainability labels are not always fully understood. As a result, consumers may have difficulty selecting sustainable products, even if they are motivated to make environmentally friendly choices [14]. Research shows that the food industry should exercise more transparency when providing sustainability information to consumers in the Marketplace [6,15].

In the context of Türkiye, the absence of legislation specifically regulating sustainability labelling creates a notable policy and implementation gap. A previous study examined whether certain labels potentially relevant to sustainability were present in Türkiye [16]. However, the use of sustainability-related labels such as animal welfare, organic production, recyclable packaging, Green Dot (ÇEVKO), Rainforest Alliance certification, local production, geographical indication, good agricultural practices, and vegan/vegetarian declarations remains voluntary and inconsistent across the market. This study is the first systematic attempt to assess both the prevalence and visibility of sustainability-related claims and logos on food labels and packaging in Türkiye, while also examining how grocery layouts may influence consumer access to and selection of more sustainable options. By highlighting current practices and gaps, this study aims to provide evidence-based guidance that may encourage producers and retailers to adopt sustainability labelling more widely and effectively.

## Materials and methods

**Fig 1:**
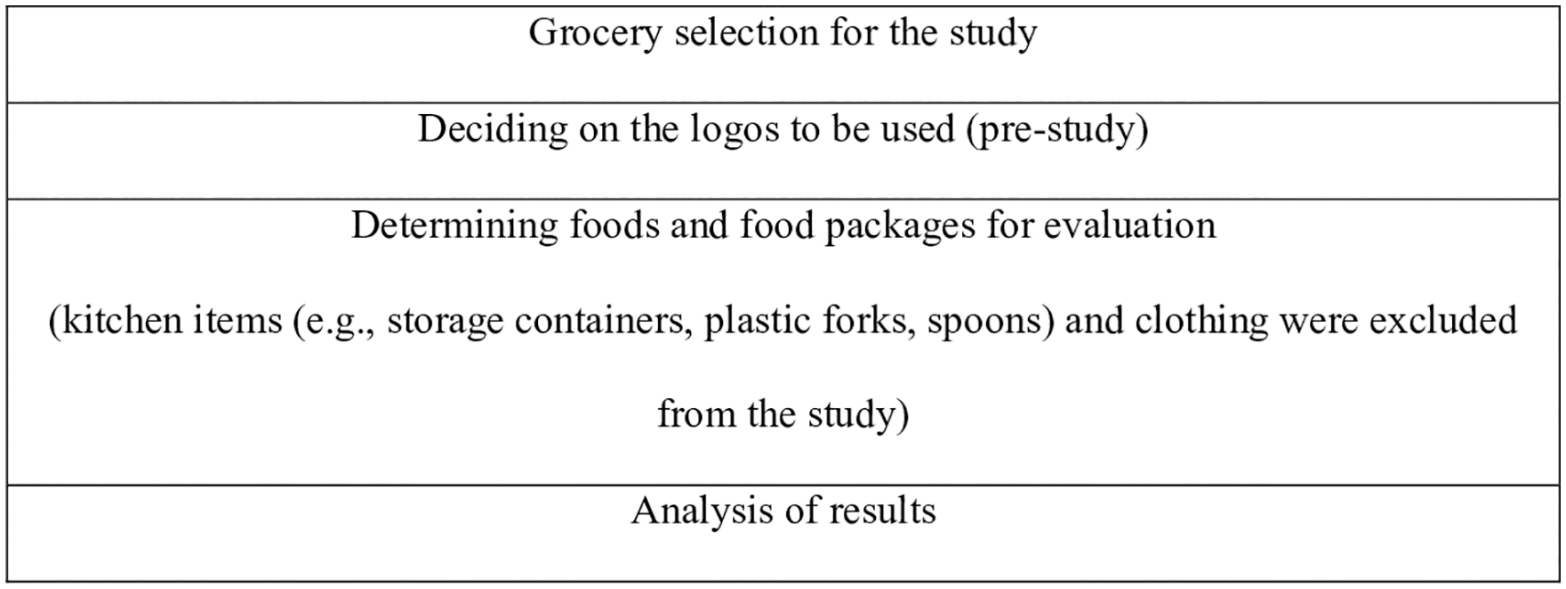
The study protocol.

The study was conducted between January 2023 and October 2023. Food packages and labels sold in grocery stores were examined within the scope of the study. The study protocol was shown at Fig 1. The suitability of the grocery for promoting environmentally sustainable behaviors was evaluated. The presence of sustainability practices in grocery was also examined. All data for this study were collected by the researchers through visits to the markets.

All procedures were approved by the Hacettepe University Non-Interventional Clinical Research Ethics Committee (Decision No: 2022/02–52, 102 dated 18.01.2022).

### Grocery selection for the study

Markets were selected based on the criterion that the company had groceries in every province of Türkiye (81 provinces). It has been determined that three grocery companies meet this criterion in Türkiye. Grocery stores were included only if they agreed to participate in the study, and all grocery companies (100%) agreed to participate in this study. The names of these groceries have been kept anonymous; their labelling is X, Y, and Z. Market names were anonymised to ensure data protection and prevent unfair competition.

While deciding on the grocery branches, 1 branch of each grocery in the 1st Region districts of Ankara, which is the 1st Region province, was included by making use of the Socio-Economic Development Ranking of Provinces/Districts Research SEGE-2017 report of the Ministry of Industry and Technology [17]. The practices, such as “presence of recycling bins” and “availability of a reusable bag incentive” that encourage consumers to make sustainable choices in grocery interior design were analysed. Sustainability-related investments of grocery companies were also determined by a questionnaire.

### Determining foods and food packages for evaluation

All food products available in the groceries were included in the assessment. However, kitchen items (e.g., storage containers, plastic forks, and spoons) and clothing were excluded from the study. The food categories were determined based on grocery store shelf layouts, given that shelf layouts play a crucial role in influencing consumer perceptions and purchasing behavior. The food categories were first created, and evaluations were made according to these categories (S1 File). In the departments of these groceries; 1) meat, poultry, fish and their products; 2) animal milks and plants based milks; 3) grains and legumes and their products; 4) fruits and vegetables; 5) snacks; 6) spices and flavourings; 7) oil and nuts; 8) beverages; 9) ready to eat foods; 10) sugars (candies) are grouped under 10 categories. In cases where some foods did not have outer packaging, such as certain fruit and vegetable, the labelling information in the respective shelf where the food is sold was analysed. Foods that consist of a mixture of several foods and can be consumed directly upon opening the packaging are classified as ready-to-eat foods.

In total, the labels and packages of 3085 foods in these 10 categories were examined. Cook et al. described three types of existing “Sustainability Labelling” such as; environmental (carbon footprint, multi-indicator scoring, sustainability claims), social (equitable trade practices, ethical treatment of workers), and animal welfare (living conditions, use of antibiotics or hormones) [18]. In Türkiye, labels that meet these definitions and are considered likely to have environmental impacts (environmental labels) have been identified as follows; recycle logo, ÇEVKO green dot logo, organic product logo, local production logo, rainforest alliance certification logo, geographically marked product logo, vegan-vegetarian logos and good farming practice logo and animal welfare logo/claims. The presence of these logos has been determined by our previous study on consumer awareness [16]. As a result of the analysis, the number of products with each sustainability indicator was calculated proportionally (logo-only, text-only, or both).

### Statistical analysis

All statistical analyses were conducted by the IBM SPSS program. The study data were expressed as mean numerical values. Quantitative data were expressed as total and percentage. The numbers of products are summed separately and categorised according to food groups. A chi-square test of independence was conducted to examine the association between food categories and the presence of various sustainability-related logos and claims.

## Results

General characteristics of the examined groceries are shown in Table 1. Their number of stores was 11.269 and their length of service is 23.7 years in Türkiye. All grocery stores have recycling bins. There were signboards next to the payment area in all groceries about reusable-bag usage. In addition to having sustainability departments, the X grocery also has energy-saving lighting in its stores and doors added to open refrigeration cabinets. In addition, 100% of its warehouses, 96% of its stores and central offices have zero waste certificates. Y grocery has been investing in renewable energy on warehouse roofs for 3 years. In addition to the 6th sustainability report published in 2023, Grocery Z has various projects, such as the Store “Energy Efficiency” and “Agriculture from Farm to Table”.

**Table 1:** General characteristics of the examined groceries.

	Number of stores	Length of service (years)	The presence of recycling bins	Availability of a reusable shopping bag incentive
X	12.000	15	Available	Available
Y	11.525	28	Available	Available
Z	10.281	28	Available	Available
Mean	11.269	23.7	100%	100%

Sustainability logos and claims observed on grocery shelves are summarized in Table 2. The recycling logo was the most prevalent, particularly on spices/flavourings (98.5%), animal and plant-based milks (98.3%), and sugars (98.0%). The ÇEVKO Green Dot was mainly observed on beverages (37.0%) and snacks (35.9%). Other labels were less common: local production (notably on sugars, 32.7%, and meat products, 31.0%), Rainforest Alliance (up to 4.5% in beverages), organic products (≤2.0%), geographical indications (≤1.6%), vegan/vegetarian logos (≤0.6%), good farming practice (12.6% on fruits/vegetables), and animal welfare (≤0.8%). Overall, 87.4% of products displayed a recycling logo, while the prevalence of other sustainability-related labels remained below 23%.

**Table 2:** Sustainability logos/claims were examined in grocery shelves.

	Total number of examined products	Recycle logo		ÇEVKO green dot logo		Organic product logo		Animal welfare logo/claims		Local production		Geographically Marked Product		Rainforest Alliance certification		Vegan- vegetarian logos		Good farming practice logo	
	n	n	%	n	%	n	%	n	%	n	%	n	%	n	%	n	%	n	%
Meat, poultry, fish and their products	261	237	90.8	19	7.3	3	1.1	2	0.8	81	31.0	0	0.0	0	0.0	0	0.0	1	0.4
Animal milks and plant-based milks	344	338	98.3	44	12.8	0	0.0	2	0.6	5	1.5	1	0.3	0	0.0	2	0.6	0	0.0
Grains and legumes and their products	251	236	94.0	16	6.4	5	2.0	0	0.0	11	4.4	2	0.8	3	1.2	1	0.4	0	0.0
Fruits and vegetables	159	32	20.1	4	2.5	0	0.0	0	0.0	3	1.9	0	0.0	0	0.0	0	0.0	20	12.6
Snacks	958	794	82.9	344	35.9	0	0.0	0	0.0	1	0.1	0	0.0	12	1.3	5	0.5	0	0.0
Spices and flavourings	202	199	98.5	37	18.3	1	0.5	0	0.0	0	0.0	0	0.0	0	0.0	1	0.5	0	0.0
Oil and nuts	151	141	93.4	17	11.3	0	0.0	0	0.0	0	0.0	0	0.0	0	0.0	0	0.0	0	0.0
Beverages	381	373	97.9	141	37.0	0	0.0	0	0.0	19	5.0	6	1.6	17	4.5	2	0.5	0	0.0
Ready to eat foods	329	297	90.3	58	17.6	0	0.0	0	0.0	3	0.9	0	0.0	0	0.0	1	0.3	0	0.0
Sugars (candies)	49	48	98.0	6	12.2	0	0.0	0	0.0	16	32.7	0	0.0	0	0.0	0	0.0	0	0.0
**Total**	**3085**	**2695**	87.4	**686**	22.2	**9**	0.3	**4**	0.1	**139**	4.5	**9**	0.3	**32**	1.0	**12**	0.4	**21**	0.7

**Fig 2:**
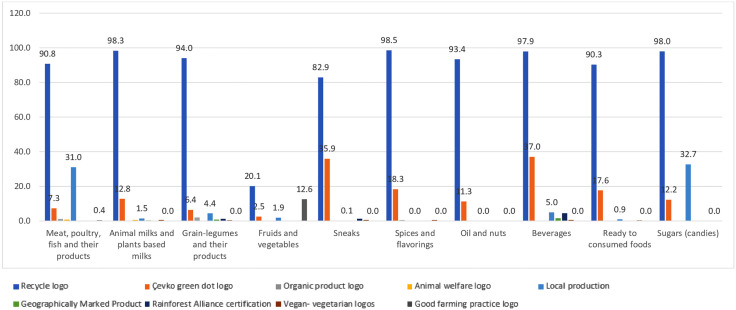
Food labels and logos were examined in grocery shelves.

Fig 2 presents the distribution of sustainability-related logos and claims across different food categories in grocery shelves. Among the identified logos, the recycle logo was the most prevalent across all categories, with particularly high frequencies in “Animal milks and plant-based milks,” “Sugars (candies),” and “Spices and flavorings.” In contrast, logos such as the Rainforest Alliance certification, animal welfare logos/claims, vegan/vegetarian logos, and good farming practice logos were rarely observed and were almost absent in most categories. Local production logos were moderately represented, especially in “Meat, poultry, fish and their products” and “Sugars (candies),” suggesting some emphasis on locally sourced goods in these groups. Organic product logos and geographically marked product labels were also observed at very low frequencies, indicating limited visibility of these sustainability-related claims in grocery stores. The analysis revealed a highly significant association between these variables (χ^2^(72)=1963.48, *p* < 0.001). This result indicates that the distribution of sustainability-related logos and claims differs substantially across food categories. In particular, the recycle logo and the ÇEVKO green dot logo were frequently observed across almost all food groups, whereas other logos (e.g., organic product, geographically marked product, vegan/vegetarian logos) appeared sporadically and were limited to specific categories. The food categories displaying the greatest number of logos on their packaging, in order, were beverages; sugars (candies); and meat, poultry, fish, and their products.

## Discussion

Meeting sustainability targets requires a reduction in diet-related environmental impacts, and food labels and claims are very important in terms of offering more environmentally friendly products to consumers. Compared to other marketing techniques, food packaging is highly effective in influencing consumers’ purchasing decisions as it communicates directly with them [19]. There is a need for a feedback mechanism between the producer and the consumer. This study aims to evaluate the relationship among consumers, producers, and intermediaries involved in offering the product. This study is the first systematic audit to assess the current situation in Türkiye regarding the use of sustainability-related labels and claims. It also examines the availability of applications that promote sustainable choices and facilitate access through grocery store interior design.

The main challenge in sustainable nutrition is the lack of social behaviour and the need for social transition [3]. Previous studies show low consumer awareness of sustainability logos and uncertainty about their prevalence on products [14,16,20]. This study also examined in-store directives, such as recycling bins and signboards promoting reusable bags, which were present in all groceries. These practices encourage waste reduction. Moreover, companies implement broader sustainability measures: Grocery X has energy-efficient lighting, closed refrigeration cabinets, and widespread zero-waste certification; Grocery Y invests in renewable energy through solar panels; and Grocery Z publishes sustainability reports and runs projects on energy efficiency and farm-to-table agriculture. Overall, all grocery chains demonstrated awareness of sustainability issues.

The shift towards food production systems with labelling and packaging supports development by improving sales and ensuring safer, better-quality products [21]. Previous studies have concluded that participants were willing to pay more for foods with less environmental impact [7,16,22]. However, it remains uncertain whether the logos and signs on food products are adequate. In this study, 87.4% of the products carried a recycling logo, followed by the ÇEVKO green dot (22.2%), local production (4.5%), Rainforest Alliance certification (1.0%), good farming practice logo (0.7%), vegan/vegetarian logos (0.4%), organic product logo (0.3%), geographically marked product (0.3%), and animal welfare claims (0.1%). Recycling logos were most common in spices and flavourings (98.5%), animal and plant-based milks (98.3%), and sugar products such as candies (98.0%). The extensive application of recycling logos may play a critical role in mitigating packaging-related waste. Additionally, achieving full and uniform coverage of such logos could enhance compliance with international environmental agreements and reinforce national commitments to sustainability. The ÇEVKO green dot, which contributes to the national Packaging Waste Recovery System [22], was mainly observed on beverages (37.0%), snacks (35.9%), and spices and flavourings (18.3%). The logo most commonly associated with sustainability on food packages in Turkey indicates the recyclability of the packaging. The earlier adoption of packaging waste recycling regulations, along with the establishment of a foundation for their evaluation, has likely contributed to greater producer awareness.

Consumption of organic food is associated with making healthy food choices, and its consumption should be encouraged [23]. In this study, the organic product logo was observed primarily on grains and legumes and their products (2.0%), meat, poultry, fish and their products (1.1%), and spices and flavourings (0.5%). The presence of the organic product logo was observed at a very low frequency. The reason for this could be that organic products are priced higher than non-organic alternatives in our country. Only a limited number of organic products are offered for sale in a small shelf of some groceries or in special sales points. Consequently, the presence of organic product logos or declarations was found to be low in this study. Increasing the visibility of these logos may alter consumer preferences, which in turn could influence producer companies. The limited presence of environmentally friendly logos on food packages contradicts the objective of establishing a sustainable production system, especially considering that labels are the primary tools influencing consumers’ food choices.

There are two logo/claims about animal welfare in Türkiye; vegan-vegetarian logos, and animal welfare logo/claim. Vegan-vegetarian logos were mostly detected in plant-based beverages (0.6%), spices and flavourings (0.5%), snacks (0.5%) and beverages (0.5%). Gerke et al. showed that the standards behind most of the producer labels were not transparent [24]. This situation is also valid for our country. Our study concluded that vegan food labels are not fully consumer-friendly and should be improved to increase transparency. The animal welfare logo was detected on meat, poultry, fish and their products (0.8%) and animal milks and plants based milks (0.6%). Quality assurance programs on animal welfare should establish clear standards and ensure accurate labelling of products to make them available to consumers [25]. Public information campaigns should prioritize increasing consumer awareness about animal welfare and expanding the grocery share of these products. Additionally, developing an animal welfare logo could be a proactive step to attract consumer attention, rather than relying solely on statements such as ‘animals are not harmed.

Logos or claims related to local production, Rainforest Alliance certification, good farming practices, and geographical indications signify origin and compliance with environmental standards [26,27]. However, literature on these labels remains limited. In this study, local production claims were mostly found on sugars (32.7%), meat and fish products (31.0%), and beverages (5.0%), while geographical indications appeared rarely on beverages (1.6%), grains and legumes (0.8%), and milk products (0.3%). Although good agricultural practices are encouraged in Türkiye, particularly in fruit and vegetable production, their low prevalence reflects insufficient disclosure by producers and retailers. Rainforest Alliance certification was observed mainly on beverages (4.5%), snacks (1.3%), and grains and legumes (1.2%), while good farming practice logos appeared on fruits and vegetables (12.6%) and milk products (0.4%). Previous research highlights challenges in consumer understanding: Annunziata et al. (2019) reported that young consumers in Southern Italy found the Rainforest Alliance logo hardest to interpret, whereas the organic food label was most trusted [14]. Futrupp et al. [13] emphasized that clearer information, consistent mandatory application, and integration with supportive policies are key to improving label effectiveness.

## Conclusions

Greater visibility and clarity of sustainability logos on food labels can enhance consumer preference. This study found that although many products met sustainability criteria, the use of logos remained limited—for instance, some plant-based items lacked vegan or vegetarian labels. As correct interpretation is vital, behavioural studies are needed to improve awareness and understanding. Moreover, consumers who are sustainability-conscious often face limited product choices, underscoring the need for producers to adopt more sustainable production systems. Overall, products with sustainability logos are expected to gain prominence in groceries, supporting sustainable development. Food companies can differentiate themselves by simplifying messages, maintaining transparent communication, and educating consumers through improved packaging, informative websites, third-party platforms, and product-specific environmental impact labels that guide quick decisions at the point of purchase.

This study highlights the need for improved visibility and consumer-friendly placement of sustainability labels, but does not provide direct evidence on the most effective methods. Future studies should examine how sustainability labels have a greater impact on consumers. A limitation of this study is the possibility of overlooking logos/claims placed on the back of packaging, as they may be challenging to realize. Logos/claims displayed prominently on the front side of food packaging are more easily perceived by consumers. Therefore, companies that prioritize consumer awareness should present information about these labelling practices in an easily understandable manner. At the same time, several limitations should be acknowledged, including the potential misclassification of logos, the use of a single-region sample, and the absence of inter-rater reliability assessment.

## Supporting information

S1 File The created food categories. (DOCX)
