# Supplementary material for: Sustainability logos and claims on food packaging and labels: The case of Türkiye
Source: PLoS One. 2025 Dec 2;20(12):e0337813. doi: 10.1371/journal.pone.0337813 (PMC12671811; doi:10.1371/journal.pone.0337813)
Supplement: S1 File — (DOCX) [file pone.0337813.s001.docx]

**S1 File.** The created food categories

| **Meat, poultry, fish and their products** | **Animal milks and plants based milks** | **Grain-legumes and their products** | **Fruits and vegetables** | **Sneaks** | **Spices and flavourings** | **Oil and nuts** | **Beverages** | **Ready to eat foods** | **Sugars (candies)** |
| --- | --- | --- | --- | --- | --- | --- | --- | --- | --- |
| Meat | Milk (animal based) | Bread | Dry fruits | Wafer | Spices | Sun flower oil | Fizzy beverages (coke, fanta, soda, mineral water) | Ready meals (such as wraps, cigköfte, mantı, meatballs, döner) | Granulated/cube sugar |
| Poultry | Milk (plant based) | Grains | Fresh fruits | Cakes | Ready to used mixes (for meat ball/chicken mixture, bouillons) sauces (vinegar, soy sauce, ketchup, mayonnaise, Napolitano sauce, pesto, tomato puree) | Corn oil | Fruit juices | Frozen products (pies, pizza, desserts) | Honey |
| Freezed meat/poultry | Cheese | Spaghetti | Freezed fruits | Cookies | Tomato paste | Olive oil | Beverages consumed cold | Powdered instant soup-noodles | Jar |
| Precooked meat/poultry | Buttermilk | Galeta, phyllo dough, lavash, sponge cake | Dry vegetables | Chocolates | Yogurt yeast, Dough yeast | Olive | Energy and sports beverages | Infant products | Grape molasses |
| Emulsified meat/poultry | Cream | Breakfast cereal and oats | Fresh vegetables | Candies | Other aroma and flavorings (currants, pine nuts, sesame, cinnamon, blue poppy, black cumin, poppy paste) | Margarines | Powdered beverages (granulated tea and coffee) | Desserts |  |
| Fish | Butter | Grain floors | Freezed vegetables | Cracker |  | Nuts and seeds |  | Fermented products |  |
| Freezed fish | Butterfat | Legumes |  | Bars |  | Sesame butters |  | Instant soup in jar |  |
| Canned fish | Kefir |  |  | Chips |  | Coconut oil |  | Dry tarhana (soup) |  |
| Offal | Yogurt |  |  | Gums |  |  |  | Chocolate spreadable |  |
|  | Other dairy |  |  | Sneaks with milk |  |  |  | Hazelnut butter |  |
|  |  |  |  | Ice creams |  |  |  | Peanut butter |  |
|  |  |  |  | Other sneaks |  |  |  | Turkish delight |  |
|  |  |  |  |  |  |  |  | Turkish fairy floss |  |
|  |  |  |  |  |  |  |  | Sucuk with walnut ( walnuts on a string dipped in starch grape molasses) |  |
|  |  |  |  |  |  |  |  | Halva |  |
